# NF-κB and Disease

**DOI:** 10.3390/ijms21239181

**Published:** 2020-12-02

**Authors:** Paola Poma

**Affiliations:** Department of Biological, Chemical and Pharmaceutical Science and Technology (STEBICEF), University of Palermo, 90128 Palermo, Italy; paola.poma@unipa.it

The role of NF-κB in all diseases characterized by an inflammatory process, from cancer to autoimmune diseases, is known, but—precisely because it is involved in many diseases—this transcriptional factor continues to attract scientific research and the new knowledge that emerges is fundamental in highlighting the therapeutic potential that this factor can have in the various diseases in which it is involved.

In this Special Issue, we have published reviews or experimental papers showing significant advances in the field of NF-κB’s role in many diseases.

Regarding research articles, we present an interesting contribution by Ko et al. about the role of NF-κB in the inflammatory process caused by CCl_4_ and resulting in an acute liver injury. The authors show that the polydeoxyribonucleotide (PDRN), an adenosine A2A receptor agonist, suppresses the secretion of pro-inflammatory cytokines and inhibits apoptosis through NF-κB inactivation. The authors conclude that PDRN could be a promising therapeutic agent for acute liver damage [1].

With a particular focus on the role of NF-κB in the inflammatory process, Lyu et al. prove that, in canine macrophages, the inflammatory responses dependent on NF-κB can be regulated by heat shock protein 70 (Hsp70). Both proteins can therefore be considered potential therapeutic targets in animal species such as the dog [2].

The first study to investigate whether the canonical, non-canonical, or atypical NF-κB activation pathways may be responsible for the higher activation of NF-κB observed in preeclamptic placentas has been published in this Special Issue. Preeclampsia (PE) is a multisystem disorder that affects 5–8% of all pregnancies worldwide, in some cases with a fatal outcome. The authors conducted the study on 268 cases (130 preeclamptic women and 138 controls) and indicate that, in the preeclamptic placentas, NF-κB activation occurs by mechanisms in which inhibitors of NF-κB such as IκBα or IκBβ may play a key role [3].

D’Ignazio et al. highlight the role of the HIF-1β isoform in the NF-κB-mediated hypoxia response. HIF-1β, in fact, controls TRAF6 expression in human cells and also in Drosophila melanogaster. Thus, given that HIF-1β is required for full NF-κB activation in cells following canonical and non-canonical stimuli, the authors conclude that HIF-1β is an important regulator of NF-κB with consequences for homeostasis and human disease [4].

Another important study on the role that many inflammatory mediators play in gamma irradiation-induced damage also focuses on the NF-κB pathway. The authors examine cell viability, reactive oxygen species (ROS), nitric oxide (NO) and glutathione (GSH) production, lipid peroxidation, DNA damage, inflammatory cytokine levels, and NF-κB pathway activation in human keratinocytes (HaCaT) and foreskin fibroblasts (BJ) after exposure to gamma radiation of 20 Gy. Contextually, they also examine the survival rate, levels of interleukin-6 (IL-6) and tumor necrosis factor alpha (TNF-α) in the blood, and p65 and phospho-p65 expression in mice after exposure to gamma radiation. In both cases, treatment with celastrol, an NF-κB pathway blocker and antioxidant, blocks the inflammatory response by reducing oxidative DNA damage and lipid peroxidation in cells, as well as preventing excessive inflammatory responses in mice, in which celastrol increases the overall survival rate [5].

Moreover, in the following article, the authors evaluate the role of NF-κB in inflammation, in particular in autoimmune disease, using relapsing–remitting experimental autoimmune encephalomyelitis (rEAE) in a mouse model that closely resembles relapsing–remitting multiple sclerosis in humans. Lunin et al. show that the reductions in the anti-inflammatory effects of thymulin (due to plasma cytokine level reduction both in the early and late stages of rEAE and decreases in NF-κB and SAPK/JNK cascade activation) are more marked when the thymic peptide thymulin is bound to polybutylcyanoacrylate (PBCA) nanoparticles. Lunin et al. show that the reduction in the anti-inflammatory effects of thymulin (both due to the reduction in the level of plasma cytokines in the early and late stages of rEAE and the decrease in cascade activation of NF-κB and SAPK/JNK) is more marked when the peptide thymus thymulin is bound to polybutyl cyanoacrylate (PBCA) nanoparticles.

Thymulin and other thymic peptides, in fact, are characterized by their very low half-life in plasma (less than 10 min) and in vivo data show that orally administered poly-butylcyanoacrylate (PBCA) nanoparticles containing the thymic hormone thymopentin had similar effects to intravenous thymopentin, but with enhanced bioavailability due to the nanoparticles [6].

The other four contributions are reviews. Ramadass et al. focus on small molecules, inhibitors of the NF-κB pathway, from cell surface receptor stimulation to nuclear signaling. The authors not only describe their clinical characteristics, but also their stage in clinical trials. This review is important because it helps to highlight the many possibilities of therapeutic choices that affect NF-κB, especially via small molecules with multiple modes of action [7].

Betzler et al. explore recent evidence regarding the association of NF-κB and immune checkpoint expression and examine the therapeutic potential of inhibitors targeting either NF-κB directly or molecules involved in NF-κB regulation, in combination with the immune checkpoint blockade. In fact, there are still a significant number of tumor patients that do not benefit from an approach consisting of the immune checkpoint blockade alone. NF-κB inhibition can increase the immune checkpoint blockade.

Leading to better patient responses, as well as different delivery systems like nanodrugs or hydrogels, the application of anti-PD-1mAb together with NF-κB inhibitors reveals promising results in relation to delivery efficacy and tolerability in preclinical models [8].

The subsequent review examines different pharmacological approaches to reducing the expression/activation of NF-κB and thus restoring chemo-sensitivity in three tumor models characterized by innate or acquired (MDR, multidrug resistance) in particular triple-negative breast cancer (TNBC), hepatocellular carcinoma (HCC), and acute myeloid leukemia (AML), given NF-κB’s key role in inducing MDR. The authors also analyze the latest scientific evidence found by other groups, the most significant clinical trials regarding NF-κB, and new perspectives on the possibility of considering this transcriptional factor a valid drug target in neoplastic diseases [9].

The last review focuses on the role of NF-κB-inducing kinase (NIK), the essential upstream kinase that regulates the activation of the noncanonical NF-κB pathway, in regulating immunity and inflammation, and presents recent studies investigating the therapeutic potential of NIK inhibitors in various diseases [10].

This Special Issue is composed of contributions that extend their scope to other topics, while always keeping NF-κB as their central focus. For this reason, this Special Issue enriches our knowledge on the role of this transcription factor in various diseases. This special issue is made up of different contributions by topic, always keeping NF-κB in the center of attention. For this reason, this special issue enriches our knowledge on the role of this transcription factor in various diseases.
